# Outcomes of autologous chondrocyte transplantation (ACT) and autologous matrix-induced chondrogenesis (AMIC) in the hip: a systematic review and meta-analysis

**DOI:** 10.1186/s13018-025-05862-5

**Published:** 2025-05-19

**Authors:** Thomas Walker, Maximilian Dewhurst, Peter Bates

**Affiliations:** 1 Centre for Trauma Science, Blizard Institute, Queen Mary University of London, 4 Newark Street, London, E1 2AT UK; 2 Department of Trauma and Orthopaedics, Frimley Park Hospital, Portsmouth Rd, Frimley, Camberley, GU16 7UJ UK; 3 Department of Trauma and Orthopaedics, Whipps Cross Hospital, Whipps Cross Rd, London, E11 1NR UK; 4 Department of Trauma and Orthopaedics, The Royal London Hospital, Whitechapel Rd, London, E1 1FR UK

## Abstract

**Background:**

Appropriate treatment of chondral lesions in the hip greatly improves symptoms and reduces the need for early joint replacement in these patients. Whilst the outcomes of Autologous Chondrocyte Transplantation (ACT) and Autologous Matrix Induced Chondrogenesis (AMIC) in the knee have been thoroughly researched, data on these treatments in the hip is comparatively limited.

**Aim:**

To evaluate the outcomes of ACT and AMIC in the hip.

**Methods:**

Following PRISMA guidelines, a literature search was performed using free text and MeSH terms relating to ACT, AMIC, and variations of these terms across 6 databases. This resulted in 506 abstracts, which were screened down to 12 papers which met the eligibility criteria. Weighted means and pooled estimates using a random effects model were used to assess the success of both procedures.

**Results:**

628 hips were identified within 12 papers. Weighted mean age 35.8 years (18–55 years), weighted mean lesion size 3.3 cm^2^ (2.2–5.1 cm^2^)., weighted mean follow-up 46.9 months (6–96 months). Improvement in mHHS was measured for both interventions, with a mean improvement of 31.1 points following ACT and 35.8 following AMIC. The pooled success rate for AMIC (99.6% [95% CI, 99.0-100.0%]) was higher than that for ACT (98.3% [95% CI, 96.4-100.0%]). All PROs assessed showed statistically significant postoperative improvements.

**Conclusion:**

Both techniques produced significant improvements from baseline. Due to the treatment characteristics, we suggest AMIC is a preferable treatment to ACT. Further research is required to assess the limitations of these procedures concerning chondral lesion size and duration of symptom improvement.

**Supplementary Information:**

The online version contains supplementary material available at 10.1186/s13018-025-05862-5.

## Background

Articular cartilage damage within the acetabulum is caused by multiple different conditions, including femoro-acetabular impingement (FAI), avascular necrosis (AVN) and developmental dysplasia of the hip, but can also occur due to trauma [1–3]. This damage can result in pain and limitation of function and can predispose the joint to osteoarthritis [3–7]. MRI has been shown to have limited effectiveness in assessing chondral lesions of the acetabulum due to its relatively thin articular cartilage and highly curved surface; but can identify possible causes of the lesions such as FAI and AVN [6, 8–11].

Hyaline cartilage in human joints has a limited regeneration capacity, and as such, the treatment of articular cartilage lesions can be challenging [3, 5, 7]. Initial treatment is conservative, and often includes analgesia (typically NSAIDs), physiotherapy and potentially corticosteroid injections. If these treatments prove ineffective then patients turn to operative therapies. Adequate treatment of acetabular cartilage damage can reduce pain and joint dysfunction, improve quality of life, reduce the risk of osteoarthritis and avoid the potential need for joint replacement [3–5].

Clinicians treating patients with symptomatic acetabular cartilage damage have a growing number of joint-preserving surgical treatments. These have been adapted from their initial use in the knee, where they have been shown to offer significant functional improvement [2, 5, 12–14]. Microfracture (MFx) was the initial treatment trialled, but newer treatments include autologous chondrocyte transplantation (ACT) and autologous matrix-induced chondrogenesis (AMIC) [5, 14–17]. Both are typically performed arthroscopically.

ACT (also referred to by some authors as autologous chondrocyte implantation) was first described by Brittberg et al. in 1994 [18], and since then, the technique has been refined and is now the an established treatment option for chondral defects > 3cm^2^ in the knee [19]. AMIC was described by Behrens as a technique to augment the traditional MFx technique in the knee, to allow treatment of larger defects > 2cm^2^ [20]. As well as use in the knee and hip, ACT and AMIC have also been adapted to allow repair of articular cartilage in the ankle and shoulder [13, 21–26].

ACT requires two surgeries, the initial one to harvest chondrocytes, which are then cultured in a lab, and a second to implant them at the site of the lesion [7, 18]. Alternatively, AMIC is performed with a single operation where MFx is augmented with the insertion of a type I/III collagen membrane onto the defect [14, 16].

Systematic reviews have been performed to compare treatments within the knee, and current evidence suggests that outcomes of AMIC and ACT are equivocal [25] In the context of talar osteochondral lesions, both AMIC and ACT have been shown to be effective treatments with favourable outcomes, although there is some evidence that modifications to technique – such as the use of fibrin glue for ACT can improve outcomes [26–28] AMIC has even been used as a revision technique for failed previous AMIC in the talus with good clinical outcomes [29]. However, studies have also shown no clinically significant benefit of AMIC over traditional microfracture [30]. Several prognostic factors for the outcomes of knee and ankle cartilage repair surgery, such as sex and increasing age [31]. Neither of these has been identified as significant within the hip previously.

Previous systematic reviews looking at the use of ACT in the hip show a paucity of data relating to all techniques for cartilage repair within the hip [1]. The majority of the published data comes from case reports and case series; no randomised controlled trials have been performed. Only one study has directly compared outcomes between ACT and AMIC in the hip [32]. O’Connor et al. performed a comprehensive meta-analysis of joint-preserving techniques for the treatment of cartilage damage in the hip [17]. Their results showed improvement in all patient-reported outcomes following ACT, but due to the heterogeneity of populations, they concluded that no inference could be made regarding the superiority of individual techniques [17].

## Aims and objectives

This systematic review aims to examine all the current evidence on the use of both ACT and AMIC in the hip to assess if one treatment provides superior outcomes, and whether specific aspects such as lesion characteristics or patient characteristics influence the clinical outcomes. This will help inform clinical decision-making in treating chondral lesions in the hip.

### Methodology

#### Review registration

This systematic review was registered on PROSPERO, ID: CRD42024516362.

## PICOS framework

### Population

Patients over the age of 18 with acetabular cartilage damage without clinical signs of osteoarthritis in the affected hip joint.

### Intervention

Autologous chondrocyte implantation of any generation, open or arthroscopic.

### Comparison

Autologous matrix-induced chondrogenesis of any type, open or arthroscopic.

### Primary outcome

Pain reduction and improved hip function, as reported by patients using standardised scoring tools.

### Secondary outcomes

Complications.

## Inclusion and exclusion criteria

*Inclusion Criteria*.

1. Studies reporting on patients over the age of 18.
2. Studies involving ACT or AMIC as an intervention for acetabular cartilage damage.
3. Studies comparing ACT or AMIC with no treatment, placebo, or alternative treatments.
4. Randomised controlled trials (RCTs), non-RCTs, prospective cohort studies, case-control studies and case series.
5. Studies with a minimum follow-up period of 1 year.

*Exclusion Criteria*.

1. Non-English language publications.
2. Studies with no full manuscript available.
3. Studies with no relevant data on primary or secondary outcomes.
4. Meta-analyses, systematic reviews, case reports, letters, reviews, feasibility studies, pilot studies, scoping studies and conference abstracts.
5. Studies reporting on patients with pre-existing osteoarthritis.
6. Studies with less than 1 year follow-up.
7. Studies with inadequate reporting of methodology.
8. Animal or laboratory studies.
9. Duplicate publications.

## Literature search strategy

A literature search was performed following the PRISMA (Preferred Reporting Items for Systematic Reviews and Meta-Analyses) guidelines for systematic reviews and meta-analyses [33]. The following databases were searched for relevant papers: PubMed; Embase; Cochrane Library; Web of Science; Scopus and Google Scholar. The search strategy included a combination of keywords, terms and MeSH terms related to “autologous chondrocyte implantation”, “acellular matrix-induced chondrogenesis”, “acetabulum” and “cartilage damage”. The search strategy was customised for each database depending on its advanced search features. The search was limited to articles published in English of the types listed in the inclusion criteria. The reference lists of these articles were also reviewed, and any further potentially eligible studies were included. As well as this, the ‘related articles’ function of PubMed was used to identify any further relevant studies. The literature search was performed on two separate occasions on the 20th of April and the 28th of May 2024.

Titles and abstracts of all identified records were assessed for concordance with the inclusion and exclusion criteria by reviewers TW and MD independently. The full article was obtained for further evaluation if it met the inclusion criteria or if there was any uncertainty. Disagreements between reviewers were resolved through discussion and consultation with reviewer PB until a consensus was made for the final list of papers to be included.

### Data extraction

Data extraction and management was performed using the Rayyan online software to collect the following information:

- Study details: Title, authors, publication year, and journal.
- Study design: RCT, non-RCT, prospective cohort, or case-control study.
- Population characteristics: Age, gender, and sample size.
- Intervention details: Type of ACT, follow-up duration, and control group.
- Outcomes: Primary and secondary outcomes.
- Methodological quality: Information on randomisation, blinding, and allocation concealment for RCTs, and confounding control for observational studies.

## Measures of treatment effect

Across the included papers, a total of 12 different clinical scoring tools were used to measure Patient Reported Outcomes (PROs), with many using several tools. Nine papers utilised the modified Harris Hip Score (mHHS); five utilised the International Hip Outcome Tool (iHOT-33); and three measured the Subjective Hip Value (SHV). Of the other nine tools used, VAS was used twice and the others were used only once, including three different variations of the EQ-5D. (Table 1). The Minimum Clinically Important Difference (MCID) has been shown to be a change of ≥11 points for both mHHS and iHOT-33 [34]. The definition of a successful surgery was taken as ‘no need for further operation to repair cartilage or conversion to Total Hip Arthroplasty (THA) within the follow-up period’.

## Data synthesis

Data synthesis was performed following the approach outlined by the Cochrane Handbook for Systematic Reviews of Interventions [35]. Aggregate study data analysis was performed using a weighted (for individual study size) random-effects proportion meta-analysis using OpenMeta[Analyst] software. Pooled estimates were calculated for the success rates of ACT and AMIC. Weighted means were calculated for age and chondral lesion size [35, 36].

### Quality assessment and Bias

The quality of the included studies was assessed with a modified version of the Coleman Methodology Score (CMS) (Appendix 1) [37]. Each study was scored for each of the 10 criteria from two parts of the grading system (part A: 7 criteria; part B: 3 criteria). The CMS evaluates the quality of the methodology, with a score ranging from 0 to 100. The higher the score, the greater the indication of a study methodology which avoids the influence of chance, errors, biases, and confounding factors [37].

Risk of bias and methodological quality was also assessed using the Methodological Index for Non-randomized Studies (MINORS) score [38]. Each point is scored 0 (not reported), 1 (reported but inadequate) or 2 (reported and adequate), with a maximum possible score of 16 for non-comparative studies and 24 for comparative studies. For this review and meta-analysis, a total score of < 8 was considered poor quality, 9–14 moderate quality, and 15–16 high quality for non-comparative studies; and < 14, 15–22, and 23–24, respectively, for comparative studies.

### Dealing with missing data

Raw data was sought for all included studies to allow for a more complete data analysis, but was not available for any. Multiple studies reported range instead of standard deviation for age and lesion size. Several methods of estimating standard deviation were assessed, but the Cochrane handbook advises against using estimated standard deviations in data analysis. As such, pooled estimates could not be calculated for age and chondral lesion size as 8/12 and 5/12 studies respectively did not report standard deviations on this data (Table 2).

## Results

### Search results

**Fig. 1:**
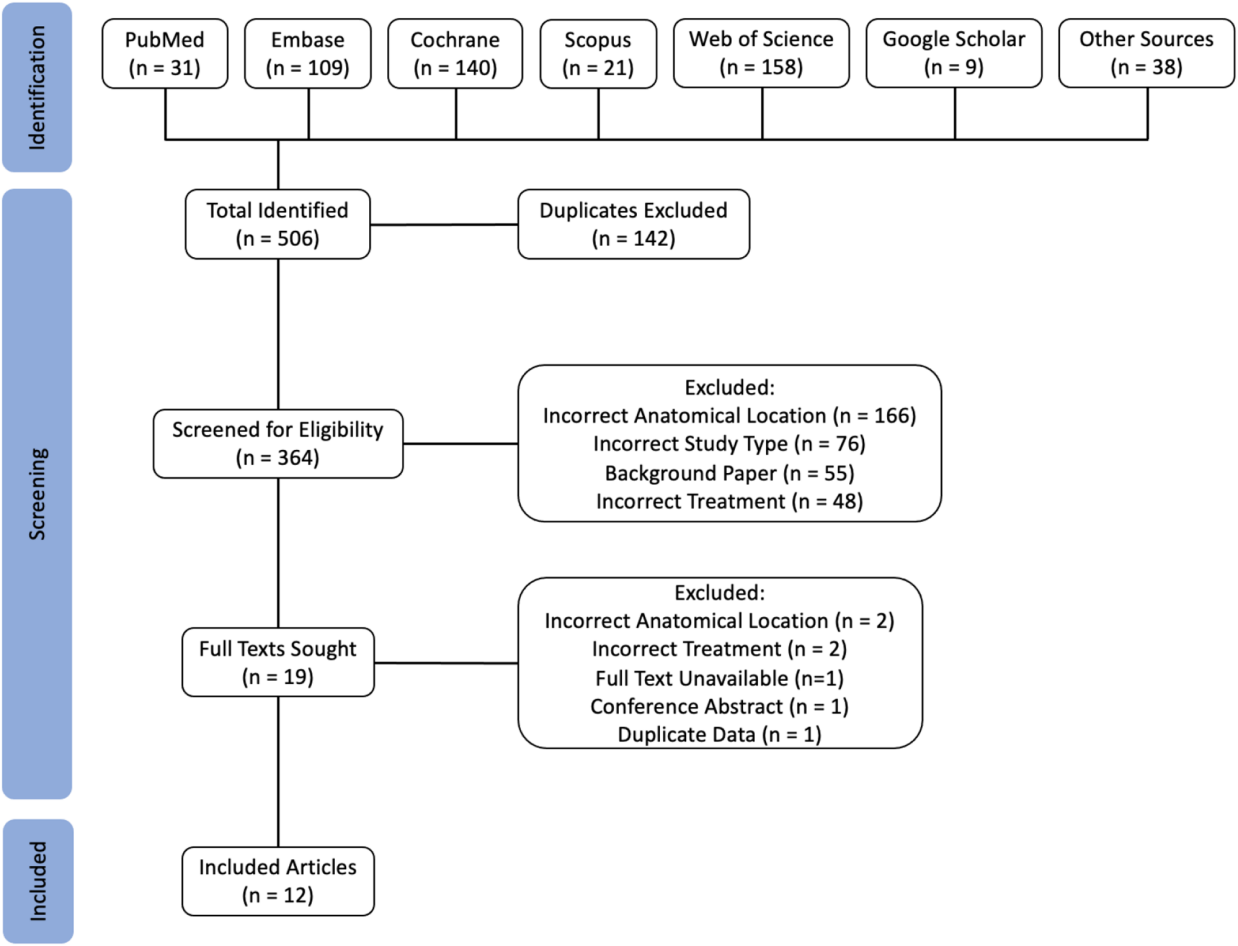
Study selection process (Prisma Diagram)

Of the 506 identified papers, 364 remained after exclusion of duplicates. A further 345 were excluded based on the title or abstract in relation to the above inclusion/exclusion criteria. Of the 19 remaining papers, one did not have an available full text, one was a conference abstract, and four were found to not meet the inclusion criteria (Fig. 1). 13 studies remained, and of these, two contained data on the same patient cohort over the same time period, but looking at different outcome measures [39, 40]. These two papers were compared, and the most useful data was kept and the other excluded.

Of the remaining 12 eligible studies, 5 contained data on ACT and 6 contained data on AMIC. One study compared treatment outcomes of ACT and AMIC [32]. This data has been displayed separately to allow a comparison of outcomes between all papers (Table 3).

**Table 1:** Clinical scoring tools used to assess patient reported outcomes throughout the included papers

Author	mHHS	iHOT-33	SHV	VAS	EQ-5D-5 L	EQ-5D-5 L VAS	EQ-5D	NAHS	OHS	COMI	UCLA	HOOS
Schroeder et al [41]	X	X	X									
Krueger et al [42]	X	X	X									
Bretschneider et al [39]		X			X	X						
Thier et al [43]		X	X				X	X				
Krueger et al [44]	X	X	X									
Mancini, Fontana [32]	X											
Briem et al [45]									X	X	X	
Thorey et al [46]	X			X								X
de Girolamo et al [47]	X											
Villarrubia et al [48]	X			X								
Fontana [49]	X											
Fontana and de Girolamo [50]	X											
Totals	9	5	4	2	1	1	1	1	1	1	1	1

**Table 2:** Chondral defect characteristics of included studies

Author	Year	Procedure	Mean Lesion Size cm^2^ ± SD (Range)	Acetabular, *n* (%)	Femoral head, *n* (%)
Schroeder et al [41]	2016	ACT	5.05 (2–6)	21 (100)	0 (0)
Krueger et al [42]	2021	ACT	5.0 (2–6)	36 (100)	0 (0)
Bretschneider et al [39]	2019	ACT	3 ± 1.4	19 (90.5)	2 (9.5)
Thier et al [43]	2017	ACT	2.21	29 (100)	0 (0)
Krueger et al [44]	2018	ACT	4.9 (2–6)	32 (100)	0 (0)
Mancini, Fontana [32]	2014	ACT	2.8 ± 0.7	26 (100)	0 (0)
		AMIC	2.9 ± 0.8	31 (100)	0 (0)
Briem et al [45]	2024	AMIC	Acetabulum 2.9 ± 0.6	8 (66.7)	4 (33.3)
			Femoral head 2.3 ± 0.6		
Thorey et al [46]	2020	AMIC	3.2 ± 0.9	62 (100)	0 (0)
de Girolamo et al [47]	2018	AMIC	3.5 (2–8)	59 (100)	0 (0)
Villarrubia et al [48]	2022	AMIC	3 (2–4)	28 (100)	0 (0)
Fontana [49]	2016	AMIC	2.9 ± 0.8	201 (100)	0 (0)
Fontana and de Girolamo [50]	2015	AMIC	3.5 (2–8)	70 (100)	0 (0)

**Table 3:** Population characteristics of included studies

Author	Year	Study Design	Procedure	Level of Evidence	Number of patients/hips	Sex M/F	Age in years ± SD (Range)	Follow up period months ± SD (Range)
Schroeder et al [41]	2016	Prospective case series	ACT	4	20/21	16/4	33 (22–49)	12.05 (6–24)
Krueger et al [42]	2021	Retrospective case series	ACT	4	36/36	31/5	32.9 (18–49)	29.9 (24–42)
Bretschneider et al [39]	2019	Prospective case series	ACT	4	21/21	17/4	32.3 ± 10 (20–53)	12
Thier et al [43]	2017	Retrospective case series	ACT	4	29/29	27/2	30.3 ± 6.9	19 (6–24)
Krueger et al [44]	2018	Retrospective case series	ACT	4	32/32	28/4	32 (18–49)	35.5 (24–49)
Mancini, Fontana [32]	2014	Retrospective Case control series	ACT	3	26/26	12/14	36 ± 9.3	60
			AMIC	3	31/31	13/18	36.4 ± 10.3	60
Briem et al [45]	2024	Retrospective case series	AMIC	4	11/12	10/1	26.8 (5.0)	74 ± 5.2
Thorey et al [46]	2020	Retrospective case series	AMIC	4	62/62	28/34	34.3 ± 5.4 (18–44)	25 (24–27)
de Girolamo et al [47]	2018	Retrospective case series	AMIC	3	59/59	27/32	39.3 (18–55)	96
Villarrubia et al [48]	2022	Retrospective case series	AMIC	4	25/28	19/6	40.5 ± 7.1 (25–55)	29 (24–48)
Fontana [49]	2016	Retrospective, non-randomised study	AMIC	4	201/201	84/117	36.4 ± 10.3	48
Fontana and de Girolamo [50]	2015	Retrospective, non-randomised study	AMIC	3	70/70	36/34	39.1 (18–55)	60

**Table 4:** Coleman methodology scores of included studies

		Part A								Part B				CMS Total
Author	Year	1	2	3	4	5	6	7	Total	1	2	3	Total	
Schroeder et al [41]	2016	4	2	10	0	5	10	10	41	7	5	5	17	58
Krueger et al [42]	2021	4	5	10	0	5	10	10	44	5	5	5	15	59
Bretschneider et al [39]	2019	4	2	7	0	5	10	10	38	7	5	0	12	50
Their et al [43]	2017	4	5	10	0	5	10	10	44	7	8	8	23	67
Krueger et al [44]	2018	4	5	10	0	5	10	10	44	7	5	5	17	61
Mancini, Fontana [32]	2014	7	5	7	0	5	10	10	44	7	5	13	25	69
Briem et al [45]	2024	0	5	0	0	5	10	0	20	7	5	13	25	45
Thorey et al [46]	2020	10	5	10	0	5	10	10	50	7	5	13	25	75
de Girolamo et al [47]	2018	7	5	10	0	5	10	10	47	7	5	10	22	69
Villarrubia et al [48]	2022	4	5	10	0	5	10	10	44	5	5	8	18	62
Fontana [49]	2016	10	5	10	0	5	10	10	50	7	5	15	27	77
Fontana and de Girolamo [50]	2015	10	5	10	0	5	10	10	50	7	5	15	27	77
	Mean	5.7	4.5	8.7	0.0	5.0	10.0	9.2	43.0	6.7	5.3	9.2	21.1	64.1
	SD	3.1	1.2	3.0	0.0	0.0	0.0	2.9	8.1	0.8	0.9	4.8	5.1	10.2

**Table 5:**
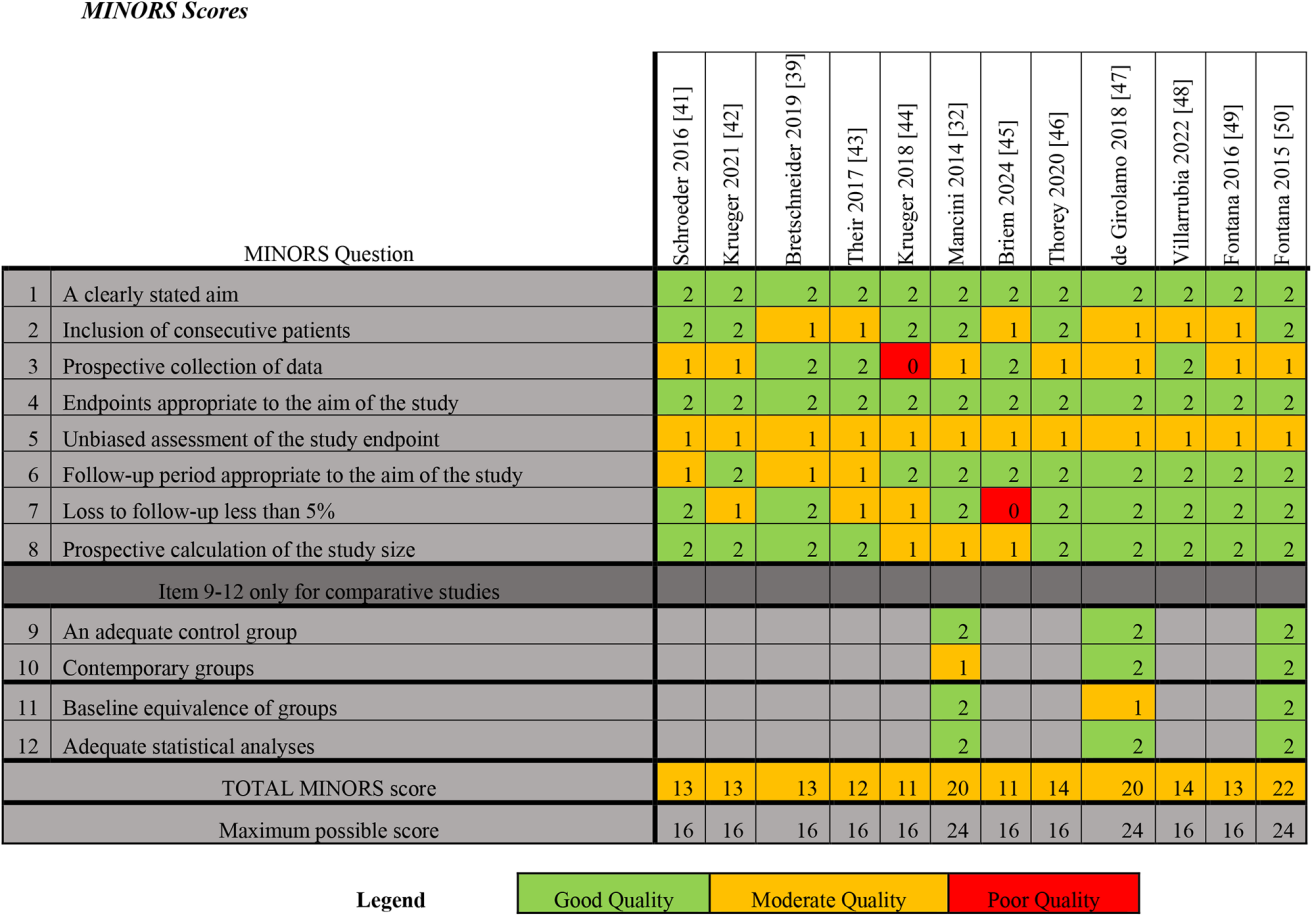
Methodological index for Non-randomized studies scores for included paper

### Quality assessment and Bias

The overall mean CMS of the included studies was 64.5 (range, 45–77) (Table 4). The mean total score of parts A and B of the CMS was 43.1 (range, 20–50) and 21.4 (range, 12–27), respectively. The main area of methodological deficiency was the study type (mean 0, range 0–0), with all of the available studies being case series. There were also deficiencies in study size (mean 6, range 0–10) and procedure for assessing outcomes (mean 5, range 0–8).

MINORS Scores for the included papers ranged from 11 to 14 for non-comparative studies, and 20 to 22 for comparative studies (Table 5). All 12 are moderate quality studies with no significant risk of bias.

### Patient cohort

Of the 628 hips included (623 patients), 165 underwent ACT and 463 underwent AMIC. 56% of patients were Male and 44% Female. The weighted mean age was 35.8 years (18–55 years), and the weighted mean lesion size was 3.3 cm^2^ (2.2–5.1 cm^2^). The proportion of patients undergoing bilateral operations was 0.8% (*n* = 5). Follow-up duration varied significantly both within and between the studies, ranging from 6 to 96 months. The weighted mean follow-up period for all the studies was 46.9 months (Table 3).

#### ACT primary outcomes

**Fig. 2:**
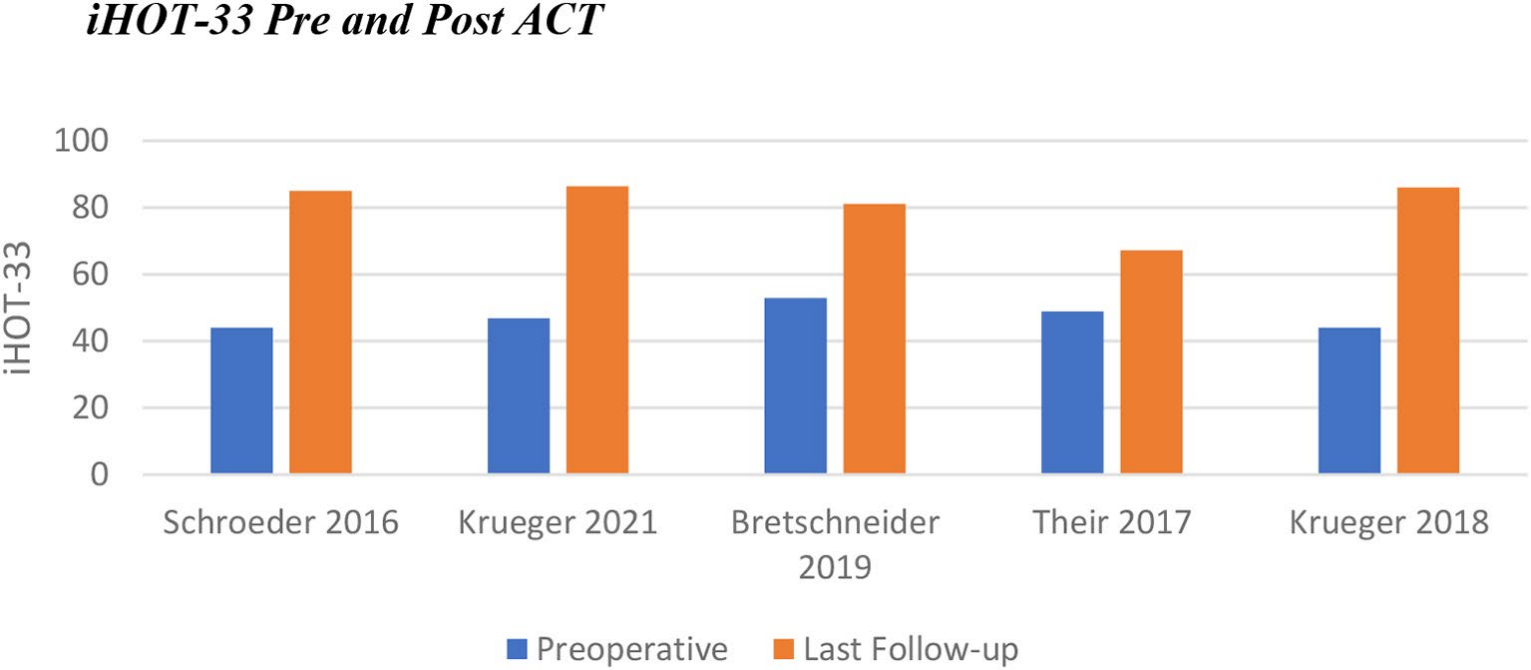
Change in modified Harris Hip Score following ACT

Preoperative average mHHS for ACT patients was 59.1 (46.5–64), with an average mHHS at last follow-up of 90.2 (85.5–92.2). The improvement for all patients (range 27–39) exceeds the MCID (Fig. 2).

**Fig. 3:**
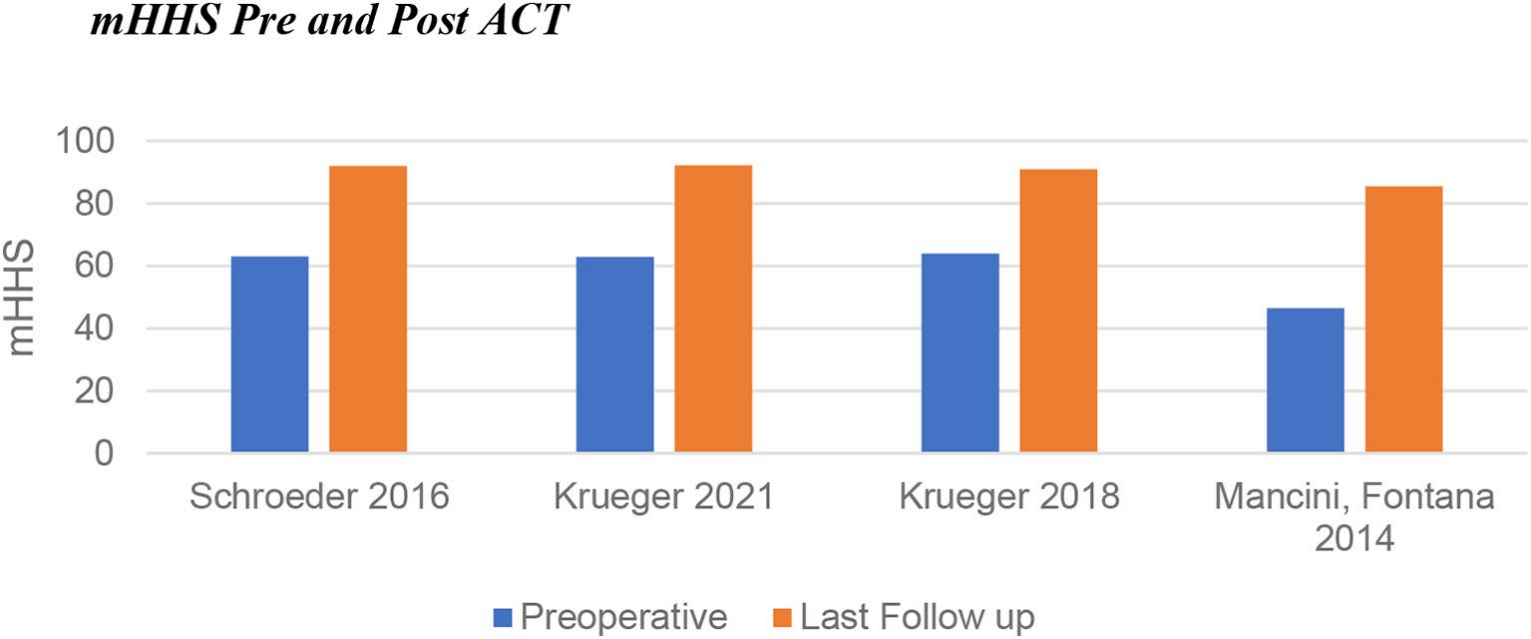
Change in iHOT-33 following ACT

iHOT-33 was reported in four of the five papers on ACT (Table 1). Average improvement was 38.3, with two papers reporting improvement of > 90% from the preoperative score [41, 44] (Fig. 3).

Multiple studies concluded that age had no impact on the preoperative or postoperative results in the mHHS and iHOT33 [41, 42, 44].

#### AMIC primary outcomes

**Fig. 4:**
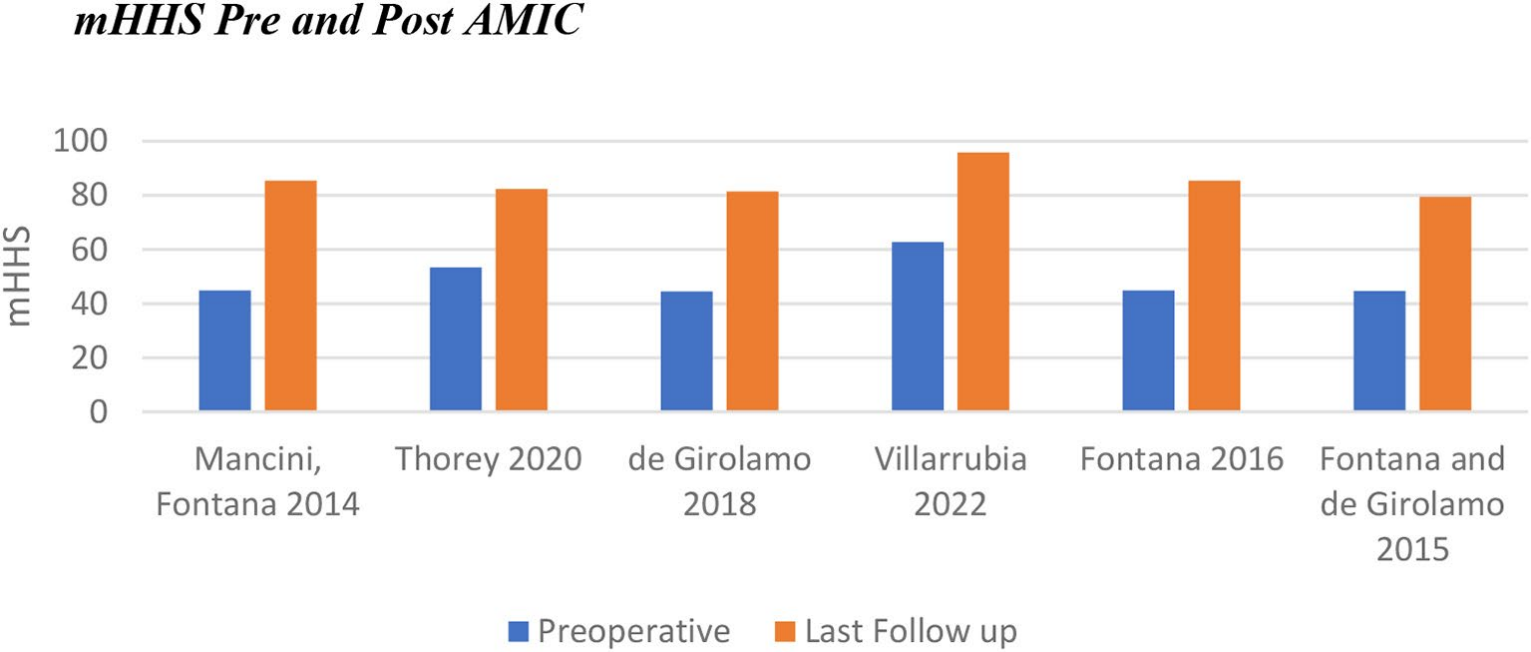
Change in modified Harris Hip Score following AMIC

Patients in the AMIC cohort had a lower average preoperative mHHS of 49.2 (44.5–62.8). Whilst the range of improvement was similar to that of ACT (29-40.6 for AMIC), the average improvement in score was greater for AMIC (35.8 compared to 31.1 for ACT). This resulted in an average mHHS at last follow-up of 85.0 (79.5–95.8) (Fig. 4).

### Outcomes/Complications

**Fig. 5:**
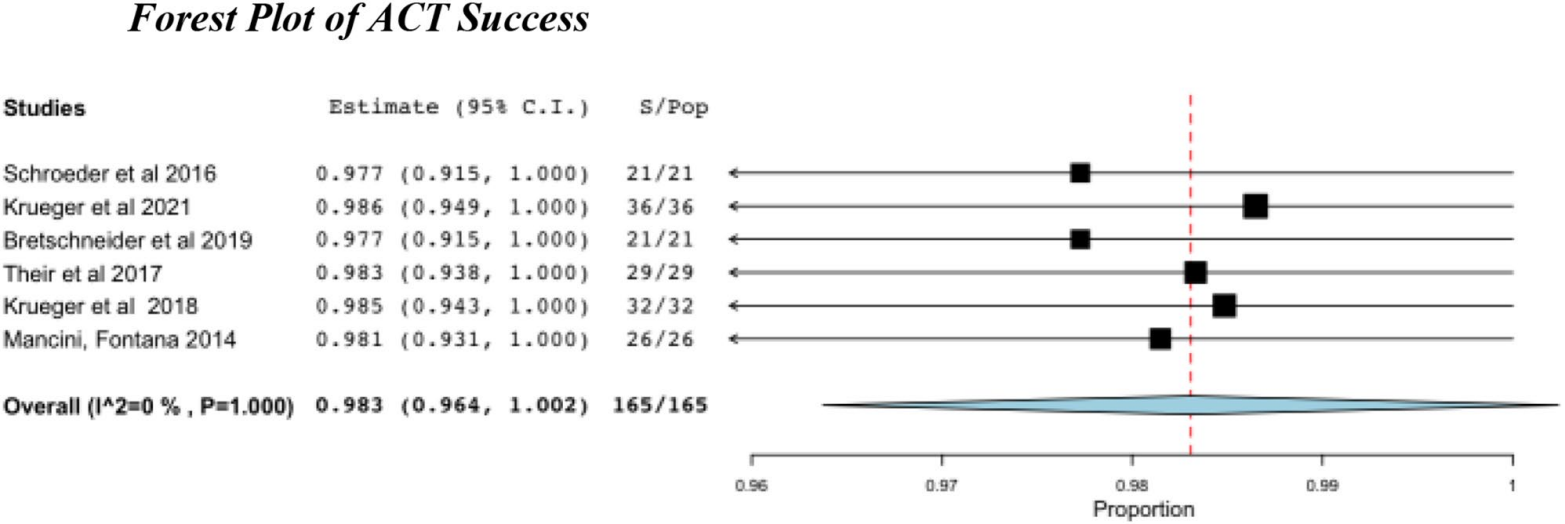
Success after ACT. S, successes; Pop, population

**Fig. 6:**
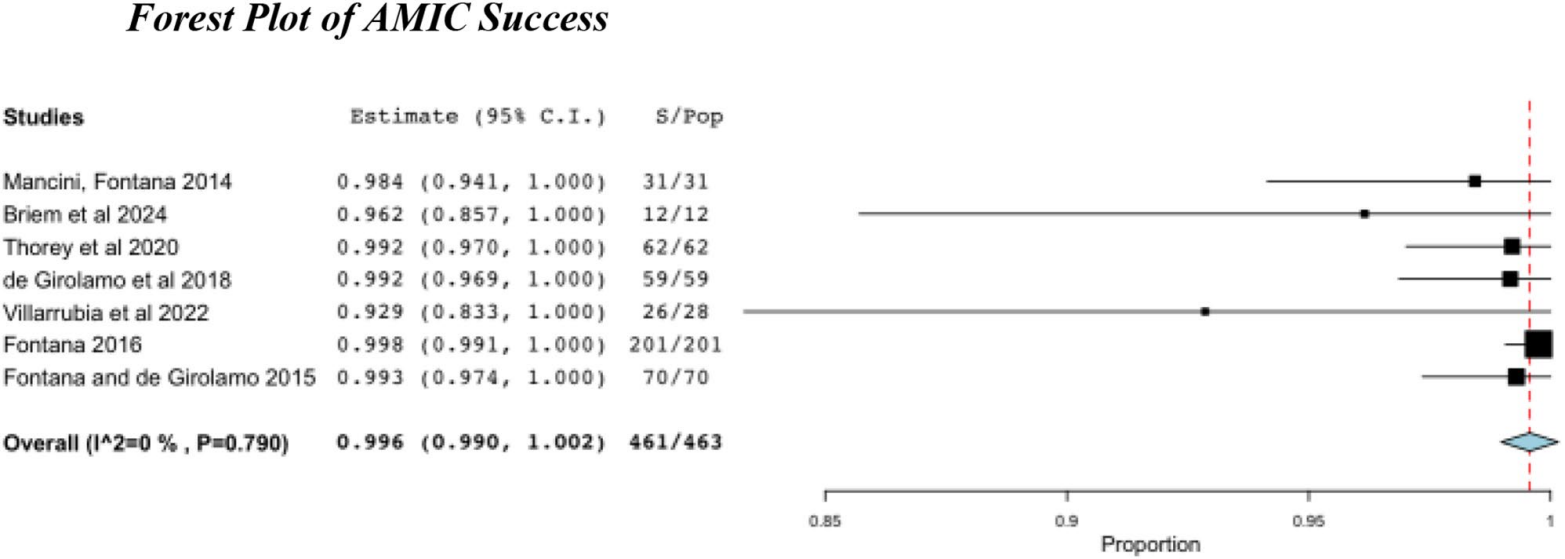
Success after AMIC. S, successes; Pop, population

The overall success or failure of either procedure was measured by the need for re-operation or conversion to THA within the follow-up period. Both ACT and AMIC showed high pooled success rates, with 98.3% and 99.6% respectively (Table 6; Figs. 5 and 6).

**Table 6:** Pooled success rates for ACT and AMIC, with conversion rates to THA

Procedure	Success Rate %	95% CI	THA %
ACT	98.3	96.4–100.0	0
AMIC	99.6	99.0–100.0	0.4

No ACT patients required conversion to THA compared to two within the AMIC cohort [48] (Table 6). Complication rates were low throughout, with only 3 complications reported across all studies (all in the same paper) [39].

Two ACT patients had failed cultivation of chondrocytes. Both chose to undergo another harvest operation and had successful implantation with no reported complications [44].

### Meta-analysis of pros

Of the 12 included studies, only one did not report PROs that were comparable to other papers [45]. For ACT, four of the six papers (66.7%) reported mHHS, and iHOT-33 was reported in five (83.3%). mHHS was the only comparable PRO reported for AMIC in six of the seven papers (85.7%) (Table 1). 100% of outcomes reported for both mHHS and iHOT-33 reported a statistically significant increase (*p* < 0.05) for both ACT and AMIC [32, 39, 41–50].

## Discussion

Only four papers collected data prospectively [39, 43, 45, 48]; and all 12 had issues with potentially biased assessment of the study endpoint. These factors along with other methodological issues meant that no papers met the overall MINORS criteria for a good quality study. Fontana 2015 was the highest quality study, with the highest MINORS and CMS scores (22 and 77 respectively) [50].

Analysis of the CMS revealed a suboptimal study design in the majority of included papers, especially regarding study size, and type of study. The restricted quality of the available studies indicates that the overall success of these interventions may potentially be biased due to prejudiced study design and outcome assessments. None of the included studies were randomised, and only 6 had populations > 40 patients (Table 3).

**Fig. 7:**
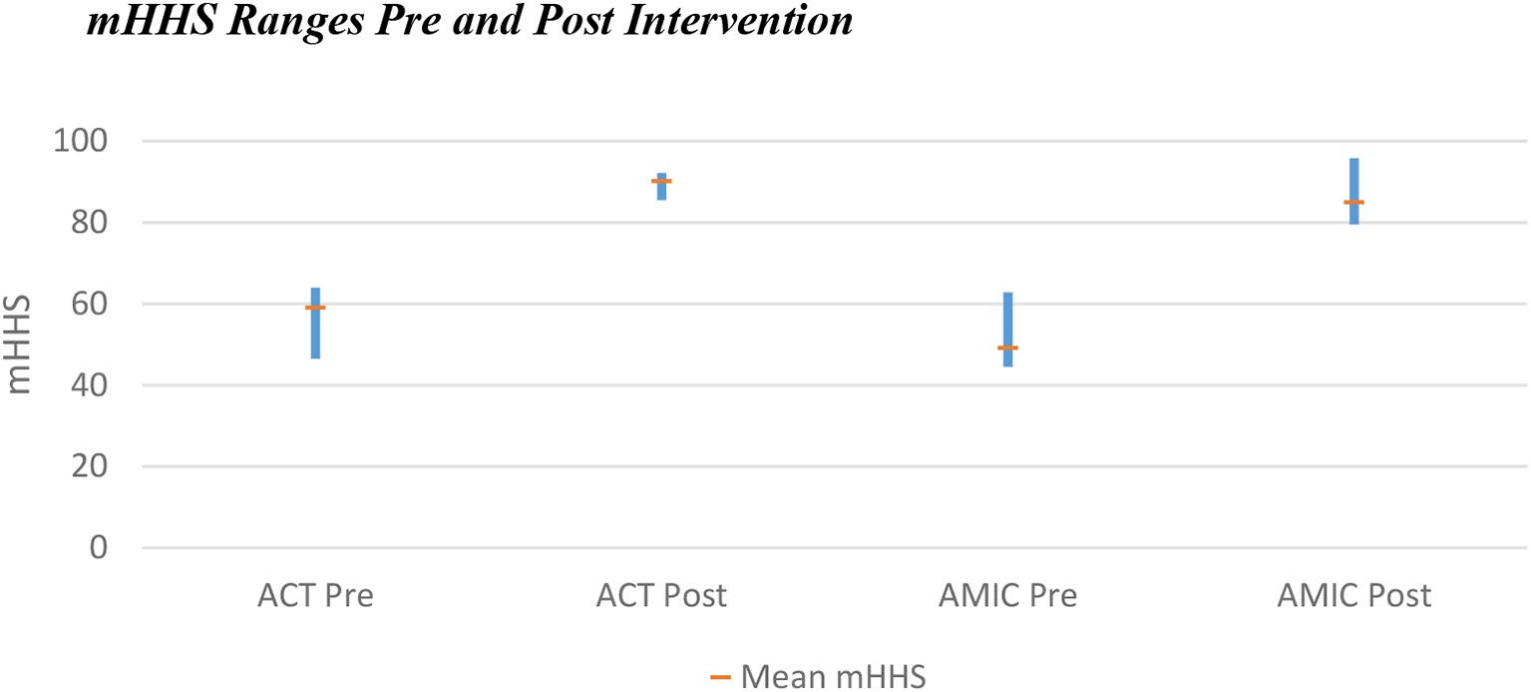
Pre and post intervention modified Harris Hip Scores including the range of means from included papers

Whilst both interventions displayed statistically significant improvements in PROs, a larger increase was noted with AMIC (Fig. 7). Heterogeneity between the populations for each intervention may have some impact on this (Table 3). The mean lesion size was larger for the ACT group, 3.9cm^2^ compared to 3.1cm^2^. The mean age was higher for patients undergoing AMIC, being 36.9 years in contrast to 32.7 for ACT. The sex distribution was also significantly different between populations: 80% male for ACT versus 47% for AMIC. None of the papers commented on the difference in outcomes between male and female patients for either intervention. In the knee, it has previously been noted that male patients undergoing ACT have better outcomes than female patients, however more recent evidence refutes this [51, 52]. No significant difference was found between male and female patients undergoing AMIC in the knee [53].

Of the included studies, only Briem et al. utilised an open approach to perform their cartilage repair. All other included papers used an arthroscopic approach [45]. As such, no conclusion can be drawn regarding the impact on outcome of an open versus arthroscopic approach specifically related to the success of the cartilage repair technique.

**Fig. 8:**
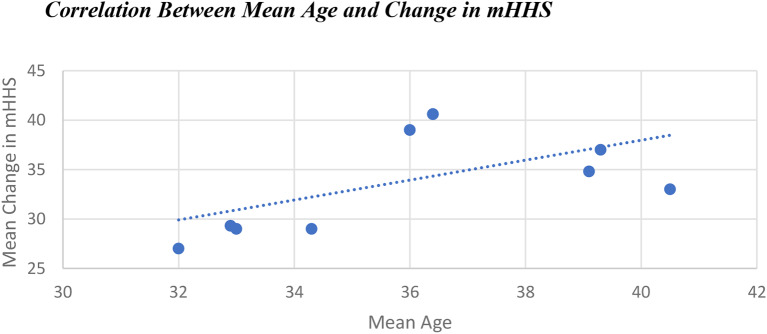
Scatter chart of mean change in mHHS against mean population age for included studies

One paper noted that older age (alongside larger cartilage defect and lower preoperative PRO) was associated with greater improvement in PRO postoperatively [39]. They themselves state that this is a controversial finding, and the findings of other studies disagreed with their conclusion [41, 42, 44]. Pooling data from the included studies does show a weak correlation between increasing age and improvement in mHSS following intervention (Fig. 8). However, there is not sufficient data reported to conclude whether this is a direct or indirect correlation, or the impact of confounding factors, such as the fact that older patients generally reported lower preoperative PROs [47, 50].

Despite the raw success rate for ACT throughout the papers being 100%, the pooled success rate using the random effect model was lower than compared to the AMIC group in which two patients underwent THA (Table 6). This is due to the smaller sample size, 165 for ACT compared to 463; and lower powered studies. As such, the data is insufficient to conclude that it has superior outcomes to AMIC.

Whilst it can be inferred from the provided data that the two AMIC patients who underwent THA within the follow-up period did so because of a lack of satisfaction with the outcome of their AMIC, this is not explicitly stated, and they may have required THA for other reasons not directly related to their initial chondral defect [48].

The complications reported (bacterial arthritis, persistent arthralgia and superficial wound healing issues) for ACT were all thought to be related to patients having multiple operations on the same site within a relatively short period of time, rather than being related to the specifics of the treatment [39]. This issue could be minimised by taking the donor chondrocytes from a different site, as was done in other papers not reporting complications [50]. However, this does add the potential issue of donor site morbidity, which was not reported in any of the included papers but has been noted by other authors [54].

The two patients undergoing ACT whose chondrocyte cultivation failed underwent a second harvesting procedure [44]. This additional operation comes with its own risks, as well as further delaying the treatment for their chondral defect. Both implantations for these patients were successful, but there was no comment on the delay that this issue caused in their treatment.

Mancini and Fontana 2014 was the only paper which directly compared the two treatments. They concluded that both procedures are valid treatments for the treatment of medium-sized chondral defects on the acetabular side of the hip and lead to long-term favourable outcomes. They did not comment on the impact of the delay in treatment for ACT whilst the chondrocytes were cultivated. Whilst their results did not show a statistically significant difference in improvement, they recommended favouring AMIC because it is a single-stage procedure and eliminates the need for specialised centres and laboratory support to cultivate cells, which reduces the total treatment time and overall cost, compared to two-stage procedures such as ACT [32].

Postoperative time was measured after the implantation operation not the harvest operation, so there may have been a further deterioration in condition for the ACT patients whilst awaiting treatment.

Donor sites and duration of time taken to culture the chondrocytes were not specified across the papers on ACT. This, alongside the lack of comment on specific donor site morbidity makes it impossible to comment on how the harvest operation impacted on PROs.

## Limitations

One limitation of this systematic review is the small number of studies (*n* = 12) that specifically reported outcomes of ACT and AMIC in the hip. Of these, multiple studies were performed at the same institutions and by the same authors, introducing a potential source of bias. Only one of the included studies was a comparison between the two treatments [32]. As such, pooled estimates demonstrated wide CIs. Of the 12 studies, only five had sample sizes > 40, limiting the power of their outcomes. None of the studies met the MINORS criteria for a high-quality study. All papers were level III or IV evidence, and the results from this analysis should be taken in the context of the level of evidence available in the published literature.

Studies included ranged over a 10-year period. For both interventions, there have been multiple iterations over this time. There is inadequate data to assess how this impacts outcomes. Concurrent procedures that were performed to treat causes of the lesions may have affected outcomes, despite the methodology of statistical analysis used.

mHHS as a measure of symptom improvement may not be sensitive enough to discern subtle changes in function in young, otherwise healthy patients; given that it is validated for assessment of functionality in elderly arthritic patients [55].

Due to missing data, such as standard deviations or confidence intervals, not all PROs from all eligible studies were included, which is another limitation. A challenge encountered in the process of data aggregation and meta-analysis was the variation of inclusion criteria used by the eligible studies. The incongruity of numerical PROs, with PROs presented graphically in one study, calls into question the validity of their results [44]. Furthermore, the lack of numerical data within studies, and lack of published raw data, limits possible meta-analysis.

## Conclusion

Both ACT and AMIC have shown to give significantly superior outcomes compared to MFx [16, 32, 50, 56]. These improvements were shown to persist, with significant improvement from the preoperative state even up to eight years postoperatively [47]. Both appear to be viable treatments for chondral defects within the hip with minimal complication rates.

Despite data from Mancini and Fontana suggesting that AMIC can be reliably extended to 4 cm^2^ defects, further research should be undertaken to look at outcomes of AMIC in patients with larger chondral lesion sizes. The main benefits of AMIC over ACT are that it is performed in a single procedure and requires less specialist equipment. The data shows that whilst low risk, the steps of ACT are each prone to their own issues with increased potential operative burden to the patient [32].

Given the available evidence, we suggest that AMIC should be the first choice treatment for symptomatic chondral lesions in the hip, given its reduced operative risk, shorter treatment time and lower overall cost. ACT remains an option for patients in whom AMIC proves ineffective or for significantly larger chondral lesion sizes. Prospective randomised controlled trials comparing ACT and AMIC are needed to confirm this recommendation [16, 32, 39].

## Electronic supplementary material

Below is the link to the electronic supplementary material.

Supplementary Material 1

## Data Availability

All data generated or analysed during this study is included in this published article and reference list.
