# Supplementary figures and images for: Outcomes of autologous chondrocyte transplantation (ACT) and autologous matrix-induced chondrogenesis (AMIC) in the hip: a systematic review and meta-analysis
Source: J Orthop Surg Res. 2025 May 19;20:483. doi: 10.1186/s13018-025-05862-5 (PMC12087195; doi:10.1186/s13018-025-05862-5)

Appendices

Appendix 1


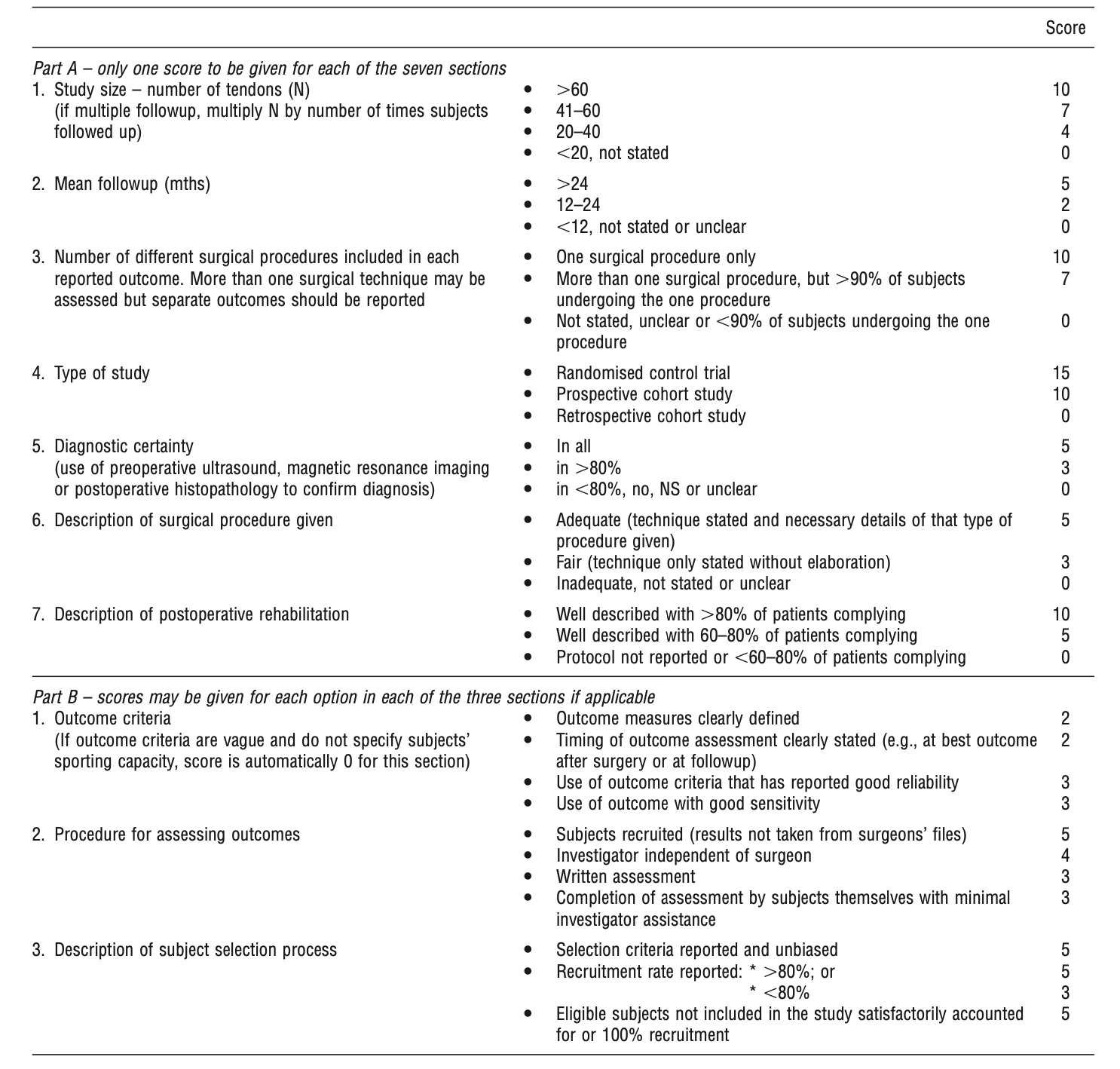

Supplement: Supplementary file 1 — Supplementary Material 1 [file 13018_2025_5862_MOESM1_ESM.docx]
